# Malondialdehyde and Antioxidant Enzymes in Maternal and Cord Blood, and their Correlation in Normotensive and Preeclamptic Women

**DOI:** 10.4021/jocmr2009.07.1252

**Published:** 2009-08-12

**Authors:** Mohd Suhail, Safia Suhail, Bharat Kumar Gupta, Vinay Bharat

**Affiliations:** a Department of Biochemistry, University of Allahabad, Allahabad-211002, India.; b City Nursing & Maternity Home Research Center, 21, Minhajpur, Allahabad-211003, India.; c Department of Biochemistry & Immunology, Subharti Medical College, S. V. S. University, Meerut- 250005, India; d Department of Pathology, Subharti Medical College, S. V. S. University, Meerut- 250005, India

## Abstract

**Background:**

An increased oxidative level and decreased antioxidant activities in the blood of preeclamptic women have been reported by us and other workers. The present study was aimed to evaluate oxidative stress in infants born to preeclamptic mothers, and to examine whether cord blood with oxygen radical disease had different total enzymatic antioxidant status than those without preeclampsia.

**Methods:**

The present study consisted of 23 normotensive (served as control) and 23 preeclamptic mothers. We compared their oxidative and anti-oxidative system in maternal and cord blood of pair-matched mother and neonate. Their blood malondialdehyde (MDA), antioxidant enzymes were determined and compared to evaluate if pro-oxidative status of preeclampsia differs from the status in cord blood of pair-matched neonate.

**Results:**

The MDA content in preeclamptic maternal plasma was significantly high (p < 0.001) compared to that of control. Interestingly, its content in preeclamptic cord blood was significantly low (p < 0.001) compared to their pair-matched maternal blood. Superoxide dismutase (SOD) activity was 8.7% higher in cord as compared to pair-matched normotensive maternal blood which was significant (p = 0.01) whereas in preeclamptic cord the level decreased significantly (p = 0.011) in comparison to pair-matched preeclamptic maternal. Glutathione peroxidase (GPx) was 16.4% higher in normotensive cord compared to maternal blood, and 7% low in preeclamptic cord compared to pair-matched maternal blood. The increase was significant (P = 0.011) in normotensive cord whereas in preeclamptic cord the decrease was insignificant (p = 0.06). Contrary to earlier reports on catalase activity, our results showed 20.97% elevation in normotensive and 16.12% increase in the preeclamptic cord blood compared to their pair-matched maternal blood. This change was significant with p = 0.01 and p = 0.017 in control and preeclamptic group respectively.

**Conclusions:**

Our results showed the significantly low MDA contents in the pair-matched cord blood and the activities of SOD, GPx and Catalase mentioned above, we conclude that the oxidative stress status is low in the blood of neonates born to preeclamptic mothers. Further studies are needed to explore strategies so that the normal levels of antioxidant vitamins are maintained to combat preeclampsia in women at high risk.

**Keywords:**

Maternal; Cord blood; Malondialdehyde; Antioxidant enzymes; Glutathione peroxidase; Glutathione reductase; Superoxide dismutase; Catalase

## Introduction

Cord blood antioxidant capacity is the result of overall intrauterine experience, making the intrauterine period crucial of the fetal development, which is the result of complex interaction among the three components of maternal-placental-fetal unit. Babies born at term to preeclamptic mothers provide a unique opportunity to have an insight into the mechanism and implications of the fetal development, secondary to intrauterine redox-status.

Our aim was to assess the relationship between lipid peroxidation and maternal/cord blood enzymatic antioxidant capacity and to examine whether cord blood with oxygen radical disease had different total enzymatic antioxidant status than those without preeclampsia (PE), a disorder of pregnancy characterized by pregnancy-induced hypertension (≥ 140 mmHg systolic and/or ≥ 90 mmHg diastolic blood pressure), new-onset proteinuria (≥ 300mg protein/day), and edema occurring in the second half of pregnancy.

Oxidative stress has been implicated in the pathophysiology of PE because it damages the maternal vascular endothelium, and there is indisputable evidence that the normal role of this cell layer is severely compromised in PE [1]. With cumulative evidences in recent years, we have recently shown that a biochemical imbalance in preeclampsia occurs with an increase of oxidative stress and lipoperoxidation and, at the same time, a deficient antioxidant protection [2, 3, 4].

Significant elevation of malondialdehyde (MDA) levels in cord blood of pair-matched preeclamptic mother have been reported [5, 6] whereas others have concluded decline in its level [7] or no significant change [8, 9], but Karabulut et al [10] inferred elevation of MDA levels both in cord and mother during preeclamptic development compared to normal pregnancy. Similarly, contradictions exist about the level of antioxidant enzymes in the cord blood of preeclamptic mothers.

Significantly decreased enzymatic activities of superoxide dismutase (SOD), glutathione peroxidase (GPx), and catalase (CAT) have been reported [6, 7, 11, 12] in cord blood of the full term newborns of preeclamptic mothers as compared to their pair-matched maternal blood. Contrary findings of increased SOD activity [8] and no change in the activity of GPx in the cord blood have also been reported [8, 9].

Inconsistency in these reports has led us to take up the present study to assess the relationship between lipoperoxidation and pair-matched cord blood antioxidant enzymatic activities; and to examine whether the maternal and cord plasma concentrations of MDA and these enzymes levels differ between preeclamptic and healthy pregnant women. Further, we aimed to investigate whether a variation or correlation in oxidant and antioxidant levels between maternal blood and fetal cord blood exists consequent to their antioxidant enzymes activities.

## Materials and Methods

### Chemicals

NADPH, Oxidized glutathione (GSSG), Glutathione Reductase (GRx), EDTA, Thiobarbituric acid (TBA) and butylated hydroxytoluene (BHT) were from Sigma Chemical Company (St. Louis, MO, USA). Other chemicals of analytical grade were obtained from either E. Merck (Mumbai, India), BDH or SISCO Chemicals (Mumbai, India).

### Subjects

In India, pregnant women are encouraged to book and to attend regular antenatal check ups. Standard antenatal care is defined as monthly visits up to 28 weeks; fortnightly until 34 weeks, and weekly visits thereafter. The present study was carried out with the prior approval of local ethical committee. The patients in our study included normal pregnant women with normal blood pressure, and preeclamptic women admitted to our hospital who had been or not under regular care and also those who were referred from private sectors or primary health centers. Twenty three normal pregnant women who served as control, and 23 severely preeclamptic patients with singleton pregnancies were selected.

Control group had two and preeclamptic group had five caesarian sections, because of prolonged labor. They gave their consent in writing and the objectives of the study were fully explained to them in detail prior to taking consent. Height and weight of the subjects were measured to calculate their body mass index (BMI). Clinical examination and history taking excluded women addicted to tobacco, patients with diabetes, ischemic heart disease, a history of stroke, kidney disorders or other conditions of known free radical etiology. The criteria for dividing women into normal and severely preeclamptic groups were set at a blood pressure of ≥ 160/110 mmHg with proteinuria, over 0.3 g protein/day, and edema.

### Sample Collection

Blood samples were collected from the mothers at delivery, cord blood was obtained immediately post partum from the umbilical vein after clamping of the cord by labor ward staff. In each case, 10 ml blood were drawn into a sodium heparin vacutainer tube for separating plasma and stored at 4 ^o^C until processed. Maternal and umbilical cord blood samples were handled identically; all samples were processed within 20 hours of sampling.

The blood samples were centrifuged at 1000 g for 15 min at 4 ^o^C, the isolated red cells were washed 4 - 5 times with 0.154 M NaCl to remove plasma and buffy coat. After the final wash, required packed red cells were lysed by hypotonic shock and different dilutions were used as hemolysates.

### Estimation of lipid peroxidation

Lipid peroxidation was quantified following the method of Jain et al [13]. Packed red cells (0.2 ml) were used for the quantification of malondialdehyde (MDA) as thiobarbituric acid reactive substances (TBARS). Aliquots of 0.2 ml were mixed thoroughly with 0.8 ml of phosphate-buffered saline (8.1 g NaCl, 2.302 g Na_2_HPO_4_, and 0.194 g NaH_2_PO_4_/L, pH 7.4) and 25 μl of butylatedhydroxytoulene (BHT, 88 mg/10 ml absolute alcohol) solution. After adding 0.5 ml of 30% trichloroacetic acid, the samples were vortexed and allowed to stand in ice for at least 2 hrs, and then centrifuged at 2000 g at 25 ^o^C for 15 min. One ml of supernatant was mixed with 75 μl of 0.1M EDTA and 250 μl of 1% thiobarbituric acid in 0.05M NaOH and placed on boiling water for 15 min. After cooling to room temperature, absorbance was measured at 532 nm and 600 nm. Absorbance at 600 nm was subtracted from absorbance at 532 nm for evaluation of MDA. BHT, an antioxidant, was added to prevent MDA formation during assay, which could result in falsely elevated TBA reactivity. The addition of BHT to standard MDA did not affect the color development with TBA. MDA contents were expressed as nmol/gHb.

### Assay of GPx (EC 1.11.1.9) activity

GPx activity was measured spectrophotometrically following the method of Paglia and Valentine [14]. The lysate was mixed with an equal volume of Drabkin’s reagent to convert all hemoglobin to the stable cyanmethemoglobin form. Exactly 0.1 ml of this mixture was added to 2.58 ml, 50 mM phosphate, pH 7.0 containing 5 mM EDTA. The following solutions were then added in turn: 100 μl, 8.4 mM NADPH, 10 μl GRx (0.3 U/ml), 10 μl, 1.125 M sodium azide and 100 μl, 150 mM GSH. The reaction mixture was allowed to equilibrate at 20 ^o^C in the cuvette of the spectrophotometer. The enzymatic reaction was initiated by addition of 100 μl, 2.2 mM H_2_O_2_. The conversion of NADPH to NADP was followed by continuous monitoring of the change in absorbance of the system at 340 nm between 2 and 4 minutes after initiation of the reaction. One unit of GPx was considered to be the amount necessary to oxidize 1 μmol NADPH/min. Activity is expressed as U/gHb.

### Assay of SOD (EC 1.15.1.1) activity

SOD activity was measured according to the method of Beutler [15]. Briefly, the reaction is dependent on the presence of superoxide anions that cause the oxidation of pyrogallol SOD which inhibits the auto-oxidation of pyrogallol, by catalyzing the breakdown of superoxide. The inhibition of pyrogallol oxidation by SOD was monitored and the amount of enzyme producing 50% inhibition was defined as one unit of enzyme activity. The assay mixture contained 1 M Tris HCl-5 mM EDTA buffer, pH 8.0, and 10 mM pyrogallol. The inhibition of pyrogallol oxidation by SOD was monitored at 420 nm on a recorder giving full-scale reading of 1 OD, the activity of enzyme was evaluated and expressed as U/gHb.

### Assay of Catalase (EC 1.11.1.6) activity

Catalase decomposes the H_2_O_2_ and forms water and molecular oxygen. H_2_O_2_ absorbs maximum light at 240 nm. When H_2_O_2_ is decomposed by catalase, the absorbance decreases. Determination of catalase activity was assayed by monitoring the rate of decomposition of H_2_O_2_ spectrophotometrically at 240 nm following the procedure of Aebi [16]. The assay mixture contained 2.0 ml of enzyme or homolysate and 1 ml, 30 mM H_2_O_2_ at 20 ^o^C with the final volume of 3.0 ml against a blank containing 1 ml, 50 mM phosphate buffer, pH 7.0, instead of substrate and 2 ml enzyme solution or hemolysate. The reaction was started by the addition of H_2_O_2_, 0.9 ml of 1 M Tris, 5 mM EDTA buffer, pH 7.0 and 0.1 ml of the sample. The decrease in absorbance was measured with a recorder at an interval of 30 seconds for 3 minutes. The value of absorbance of the reference was subtracted from that of the test cuvette before units of activity were calculated. The activity of catalase was evaluated and expressed as kU/gHb.

### Assay of GRx (EC 1.8.1.7) activity

The main reagent was prepared by combining 18 ml of KH_2_PO_4_ buffer 139 mM, 0.76 mM EDTA, pH 7.4, and 2 ml of 2.5 mM NADPH. The sample (20 μl of 1: 20 hemolysate + 20 μL of KH_2_PO_4_ buffer), 220 μl of the main reagent and 5 μ of FAD 0. 315 mM + 10 μl of KH_2_PO_4_ buffer were added to the cuvette and the absorbance at 340 nm was monitored for 200s (step A). Then 30 μl of GSSG 22mM + 10 μl of KH_2_PO_4_ buffer were added to start the reaction and the absorbance was followed for 175 s (step B). The final reaction volume was 315 μl. The difference in absorbance per minute between steps B and A was used to calculate the enzyme activity. The unit is μmol of NADPH oxidized /min and the GRx activity is evaluated and expressed as U/gHb [17].

### Hemoglobin estimation

The method of Tentori and Salvati [18] was employed for hemoglobin estimation. Hemoglobin content of the sample was measured using cyanmethemoglobin method by mixing 20 μl of blood and 5 ml of (1: 251 diluted) ferricyanide reagent, K_3_Fe (CN)_6_, 200 mg; KCN 50 mg; KH_2_PO_4_ 140 mg; appropriate amount of detergent Triton X-100 dissolved and raised to one liter, pH 7.4, and allowing to stand for at least 3 min. Afterwards, absorbance was read at 540 nm using water as blank.

### Statistical analysis

SPSS version 15.0 for Windows (SPSS Inc., Chicago, IL, USA) software package was used to analyze the data and to obtain the box-plots for various parameters. The results were statistically analyzed using paired-samples t-test to compare both maternal/cord blood of normotensive pregnant and preeclamptic patients groups. The t-test statistical significance was set at P ≤ 0.05. Values were expressed as Mean ± Standard Deviation.

## Results

We evaluated the quantum of enzymatic antioxidant defense in maternal and cord blood of two groups of pair-matched maternal and neonates. Control group consisted of 23 uncomplicated, normotensive-pregnancies and the second group included 23 severely preeclamptic patients, all singleton pregnancies. Control group had two, whereas preeclamptic group had four caesarian sections, because of prolonged labor. The clinical characteristics of these groups are shown in Table 1. The activities of SOD, GPx, GRx and Catalase are summarized as mean ± standard deviation in Table 2.

**Table 1:** Demographic and clinical characteristics of normotensive (control) and severe preeclamptic subjects

Parameters	Normotensive control group	Severely preeclamptic group	P*-value
Number of maternal/neonatal pairs	23	23	
Maternal age (years)	29.1 ± 6.9	26.8 ± 7.2	0.275
BMI at delivery (kg/m^2^ )	22.1 ± 2.5	21.4 ± 2.8	0.376
Gestational age (weeks)	33.8 ± 3.9	32.2 ± 4.8	0.221
BP at delivery, systolic (mm/Hg)	109.8 ± 12.9	164.5 ± 13.6	<0.0001
BP at delivery, diastolic (mm/Hg)	65.6 ± 11.4	111.4 ± 14.2	<0.0001
Pulse rate (beats/min)	71.4 ± 1.6	70.8 ± 2.4	0.324
Proteinuria (g/day)	Nil	1.24 ± 0.86	/
Edema	Nil	++ in all cases	/

Values are expressed as mean ± SD; BMI- body mass index; BP- blood pressure, P*= Two-Samples t-test probability

**Table 2:** Concentrations of malondialdehyde and activities of various antioxidant enzymes in pair-matched normotensive (control) and severely preeclamptic maternal and cord blood

Parameters	Normotensive maternal (n = 23)	Normotensive cord (n = 23)	preeclamptic maternal (n = 23)	preeclamptic cord (n = 23)
Malondialdehyde (MDA) (nmol/gHb)	9.02 ± 2.36 (Cl*: 8.008 to 10.04)	7.36 ± 1.62 (Cl*: 6.66 to 8.06)	11.98 ± 1.83 (Cl*: 11.19 to 12.77)	8.02 ± 1.05 (Cl*: 7.56 to 8.47)
Superoxide dismutase (U/gHb)	1135.95 ± 100.28 (Cl*: 1092.58 to 1179.32)	1235.17 ± 119.07 (Cl*: 1183.68 to 1286.66)	921.92 ± 117.54 (Cl*: 871.09 to 972.74)	842.16 ± 81.24 (Cl*: 807.03 to 877.29)
Glutathione peroxidase (U/gHb)	15.21 ± 3.04 (Cl*: 13.9 to 16.53)	17.71 ± 2.27 (Cl*: 16.73 to 18.69)	13.92 ± 1.36 (Cl*: 13.33 to 14.51)	12.95 ± 1.89 (Cl*: 12.14 to 13.77)
Glutathione reductase (U/gHb)	11.24 ± 3.03 (Cl*: 9.93 to 12.54)	10.98 ± 2.90 (Cl*: 9.97 to 12.23)	8.98 ± 2.44 (Cl*: 7.93 to 10.04)	8.85 ± 2.46 (Cl*: 7.79 to 9.92)
Catalase (kU/gHb)	98.21 ± 21.17 (Cl*: 89.05 to 107.36)	118.80 ± 25.81 (Cl*: 107.64 to 129.96)	124.70 ± 17.48 (Cl*: 117.14 to 132.26)	144.8 ± 34.99 (Cl*: 129.67 to 159.93)

Values are expressed as mean ± SD; Cl* = 95% Confidence Interval for mean

**Figure 1:**
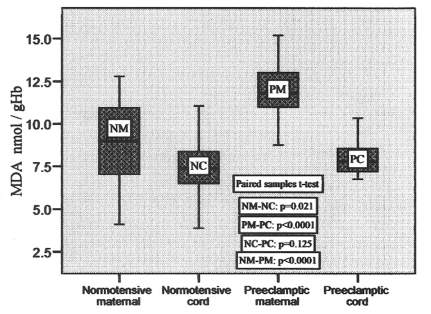
MDA concentrations in pair-matched normotensive and preeclamptic maternal and cord blood.

The MDA contents varied from 4.12 to 12.79 nmol/gHb in normotensive maternal and 3.89 to 11.06 nmol/gHb in normotensive cord, whereas it varied from 8.75 to 15.19 nmol/gHb in preeclamptic maternal and 6.75 to10.34 nmol/gHb in preeclamptic cord. Their mean values are mentioned in Table 2. The MDA content in preeclamptic maternal blood was found to be 32.8% higher than that of control. There was no significant difference (p = 0.021) between control maternal and cord blood levels. The MDA content in preeclamptic maternal plasma was significantly high (p < 0.001) compared to that of control. However, its content in preeclamptic cord blood compared to their pair-matched maternal blood was significantly low (p < 0.001). The variations in its contents in pair-matched normotensive and preeclamptic maternal as well as cord blood have been shown in Figure 1.

**Figure 2:**
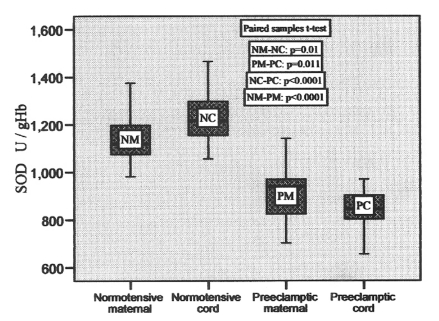
SOD activities in pair-matched normotensive and preeclamptic maternal and cord blood.

The activity of superoxide dismutase ranged from 980.73 to 1375.65 U/gHb, 1055.59 to 1465.97 U/gHb, 702.05 to 1141.71 U/gHb and 655.69 to 970.12 U/gHb in normotensive maternal, pair-matched cord, preeclamptic maternal and pair-matched cord, respectively, with the mean values given in Table 2. It was found to be 8.7% higher in cord as compared to pair-matched normotensive maternal blood which was significant (p = 0.01), whereas in preeclamptic cord the level decreased significantly (p = 0.011) in comparison to pair-matched preeclamptic maternal. The variations in its levels in pair-matched normotensive and preeclamptic maternal as well as cord blood are shown in Figure 2.

**Figure 3:**
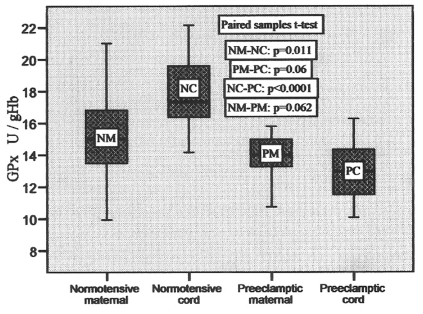
GPx activities in pair-matched normotensive and preeclamptic maternal and cord blood.

Glutathione peroxidase (GPx) was found to vary from 9.94 to 21.02 U/gHb, 14.15 to 22.14 U/gHb, 10.75 to 15.80 U/gHb, 10.08 to 16.28 U/gHb in normotensive maternal, pair-matched cord, preeclamptic maternal, pair-matched cord, respectively, with their mean values mentioned in Table 2. It was 16.4% higher in normotensive cord compared to maternal blood and 7% low in preeclamptic cord compared to pair-matched maternal blood. The increase was significant (P = 0.011) in normotensive cord, whereas in preeclamptic cord the decrease was insignificant (p = 0.06). The variations in activities in pair-matched normotensive and preeclamptic maternal as well as cord plasma are shown in Figure 3.

**Figure 4:**
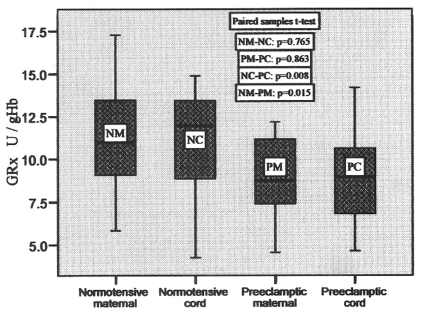
GRx activities in pair-matched normotensive and preeclamptic maternal and cord blood.

In control group, the glutathione reductase (GRx) activity ranged from 5.84 to 17.28 U/gHb in maternal blood and 4.24 to 14.89 U/gHb in cord blood, whereas in preeclamptic group it varied from 4.55 to 12.18 U/gHb in maternal and 4.65 to 14.20 in cord blood with the mean values shown in Table 2. The box-plots of these values are shown in Figure 4, which represent these variations clearly with no significant difference between maternal and pair-matched cord blood in control (p = 0.765), and preeclamptic group (p = 0.863). But, the difference in the mean values of maternal and cord blood of both groups was significant (p = 0.015 and p = 0.008, respectively). There was 20.1% decrease in preeclamptic maternal blood and 19.4% decrease in the GRx activity in preeclamptic cord blood when compared to their respective normotensive maternal and cord blood.

**Figure 5:**
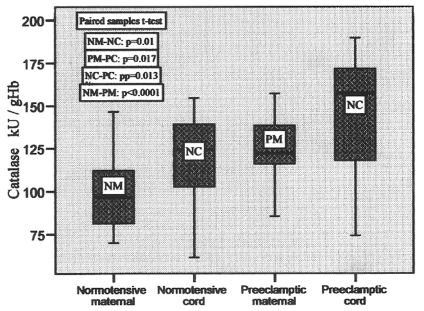
Catalase activities in pair-matched normotensive and preeclamptic maternal and cord blood.

Catalase activity was found elevated in the cord blood of both the groups. There was 20.97% elevation in normotensive and 16.12% increase in the preeclamptic cord blood compared to their pair-matched maternal blood. This change was significant with p = 0.01 and p= 0.017 in control and preeclamptic group respectively. These changes are represented in the form of box plots in Figure 5. The activity ranged from 69.99 to 146.54, 61.43 to 154.73, 85.46 to 157.23 and 74.25 to 189.58 in normotensive maternal, cord, preeclamptic maternal and cord blood, respectively, with the mean values shown in Table 2.

## Discussion

The present study investigated maternal plasma enzymatic antioxidant levels during normal and complicated preeclamptic pregnancies and their relationships with neonatal antioxidant levels. Cumulative evidences in recent years show that a biochemical imbalance in preeclampsia occurs with an increase of oxidative stress and a deficient antioxidant protection [2, 3, 4]. Indeed, free radicals released from the poorly perfused fetoplacental unit initiate lipid peroxidation by attacking polyunsaturated fatty acids in cell membranes, converting them to lipid peroxides and to a variety of secondary metabolites. Uncontrolled peroxidation alters membrane fluidity and permeability, therefore the lipid peroxides and their secondary metabolites, such as malondialdehyde (MDA, a good indicator of oxidant forces formed at a primary site), are then transported through the circulation by lipoproteins, causing damage at distant tissues.

In the preeclamptic pregnancy, the oxidant/antioxidant balance of normal pregnancy is skewed to favor oxidants at the expense of antioxidants. Antioxidant defense systems include the chain-breaking antioxidants, such as vitamin C and vitamin E, and the antioxidant enzymes. Lipid-phase chain-breaking antioxidants, the most important of which is probably vitamin E [19], scavenge radicals in membranes and lipoprotein particles and are central to the prevention of lipid peroxidation. Aqueous-phase chain-breaking antioxidants directly scavenge radicals present in the aqueous compartment. Vitamin C or ascorbate is the most important aqueous phase chain-breaking antioxidant [20]. It is well established that there is synergy between vitamins C and E. This interaction between vitamin C and vitamin E has been confirmed in vivo by workers [21], who have reported that supplementation of healthy adults with ascorbic acid increases ascorbic acid and lipid-standardized alpha-tocopherol levels in plasma, and that supplementation with alpha-tocopherol is associated with increased plasma ascorbic acid concentration, as well as improved vitamin E status. Our recent reports [22] showed significantly high vitamin C levels in the cords of both normotensive as well as preeclamptic mothers, which showed retention of comparatively elevated antioxidant capacity in the cords.

Our results showed 32.8% higher MDA contents in preeclamptics compared to normotensive maternal plasma. Earlier, it has been reported [23] that preeclamptic placenta contains higher MDA than those from normal pregnancies. Our previous reports [3, 4] have shown significant elevation of MDA or TBARS during the development of PE. This may result in a greater potential for endothelial damage ultimately leading to enhanced diastolic pressure [20] which further aggravates the condition of preeclamptic patients. Enhanced ROS in turn can oxidize many other important biomolecules including erythrocyte membrane phospholipids. The interesting finding of the present study is 33.1% lower level of MDA concentrations in the cord plasma as compared to maternal plasma in preeclamptics. Our present findings on MDA concentrations are consistent with those of Orhan et al [7].

Superpxode dismutase (SOD), Catalase, GRx, and glutathione peroxidase (GPx) are important parts of the defense system. SOD protects and revitalizes cells and reduces the rate of cell destruction. It neutralizes some of the most dangerous free radicals, the superoxide radicals, before they can wreak havoc on the body. Superoxide generation also perpetuates oxidative stress and lipid peroxidation through the oxidation of mitochondrial iron-sulphur clusters such as aconitase, which subsequently stimulate membrane phopholipid peroxidation by alkoxyl radicals [24]. SOD is an important antioxidant enzyme having an antitoxic effect against super oxide anion and catalyses the reaction in which superoxide radicals are converted to H_2_O_2_ and O_2_. It decreases superoxide anion concentration in the vascular cell [25], a mechanism that could counteract the development of hypertension. In normotensive cord, SOD increased 8.7% compared to maternal blood, but in preeclamptic cord it decreased to 8.65% as compared to their pair-matched maternal blood, and both the alterations were significant, p = 0.01 and p = 0.011, respectively. Our results pertaining to SOD activity in preeclamptic cord blood are in harmony with the reports of others [6, 7, 11, 12].

The principal function of GPx is to protect against damage from the endogenously produced hydroxyperoxides and to catalyze the reduction of hydroxyperoxides by glutathione. Catalase promotes the conversion of hydrogen peroxide, a powerful and potentially harmful oxidizing agent, to water and molecular oxygen. It also uses H_2_O_2_ to oxidize toxins such as phenols, formic acid, formaldehyde and alcohols. Catalase, along-with SOD and GPx controls the levels of oxygen-derived free radicals in cells. During our present study the alteration in GPx activities was not significant (p = 0.06) between preeclamptic maternal and cord blood. However, in control group there was 16.4% increase in GPx activity in cord blood as compared to maternal group which was significant (p = 0.011). There was no significant change in the activity of GRx between maternal blood and pair-matched cord blood of control and preeclamptic group (p = 0.765 and p = 0.863, respectively). Interestingly, the activity of catalase was found to enhance very significantly in cord blood of normotensive and preeclamptic groups as compared to their pair-matched maternal blood (21% and 16% increase respectively ), which show its compensatory regulation in response to increased oxidative stress. The significant elevation in preeclamptic catalase activity shows the protective effect of this enzyme, which protects the cells from the accumulation of H_2_O_2_ by dismutating it to form water and oxygen by using it as an oxidant in which it works as a peroxidase [26].

In conclusion, we hypothesize the elevated oxidative stress in preeclampsia, our results showed decreased SOD, GRx and GPx activities, which failed to control higher oxygen free radical produced therein, this is consistent with our previous report [3]. However, no significant decrease was found in the activities of GPx and GRx in their pair-matched cord blood. Increased activity of catalase may be a compensatory regulation in response to increased oxidative stress. Increased catalase activity in the pair-matched cord blood could be interpreted as an effort to counteract the overproduction of reactive oxygen species and provide a relief to enhanced oxidative damage in preeclampsia. From our findings of significantly low MDA contents in the pair-matched cord blood, we hypothesize that antioxidant capacity of cord blood is sufficient and placental barrier is adequate, to shield the fetus from the oxidative injury due to higher oxidative stress of preeclamptic mother. Thus, we conclude that the oxidative stress status is low in the blood of neonates compared to its level in the pair-matched preeclamptic mothers. Further studies are needed to explore strategies so that the normal levels of antioxidant vitamins are maintained to combat preeclampsia in women at high risk.
